# Cancer genomics: why rare is valuable

**DOI:** 10.1007/s00109-015-1260-8

**Published:** 2015-02-14

**Authors:** Farzad Jamshidi, Torsten O. Nielsen, David G. Huntsman

**Affiliations:** 1 Genetic Pathology Evaluation Centre, University of British Columbia, Vancouver, BC Canada; 2 Department of Pathology and Laboratory Medicine, University of British Columbia, Vancouver, BC Canada

**Keywords:** Next-generation sequencing, Formefruste, Rare tumors, Genomics

## Abstract

Rare conditions are sometimes ignored in biomedical research because of difficulties in obtaining specimens and limited interest from fund raisers. However, the study of rare diseases such as unusual cancers has again and again led to breakthroughs in our understanding of more common diseases. It is therefore unsurprising that with the development and accessibility of next-generation sequencing, much has been learnt from studying cancers that are rare and in particular those with uniform biological and clinical behavior. Herein, we describe how shotgun sequencing of cancers such as granulosa cell tumor, endometrial stromal sarcoma, epithelioid hemangioendothelioma, ameloblastoma, small-cell carcinoma of the ovary, clear-cell carcinoma of the ovary, nonepithelial ovarian tumors, chondroblastoma, and giant cell tumor of the bone has led to rapidly translatable discoveries in diagnostics and tumor taxonomies, as well as providing insights into cancer biology.

## Impact of study of rare tumors

The history of biomedical research has repeatedly shown that with the advent of new methodologies, the study of rare, but clinically well-defined disease entities has led to the generation of a disproportionate amount of knowledge. For instance in the eighteenth century, the peculiar scrotal skin cancers of adolescent males with a history of occupation as chimney sweeps led Percivall Pott to describe one of the earliest associations of workplace hazards and cancer [1]. His study shed light on the potential role of environmental factors in cancer formation and helped lay the foundations for the science of epidemiology. Another example happened in 1961 when Sir Anthony Epstein came across D.P. Burkitt’s description of an unusual new children’s cancer in Africa with a geographic distribution related to certain rainfall and temperature patterns [2]. Epstein thought of a possible climate-dependent vector and initiated a study, during a time of great advancements in molecular biology and virology, that led to the discovery of the Epstein Barr virus, the first proven human cancer virus [3]. Retinoblastoma was another relatively rare condition that led to the conceptualization of tumor suppressor genes. The comparison of kinetics of unilateral sporadic versus bilateral familial cases led Alfred Knudson Jr. to describe the two-hit hypothesis, which revolutionized cancer biology [4].

It has long been clear that mutations play a critical role in the development of cancer. However, many common cancers are both biologically and clinically complicated and their mutational landscape reflects such complexity [5, 6]. On the other hand, some perhaps more obscure tumors, especially ones affecting younger patients and specific sites, reveal simpler genomic landscapes with characteristic mutations that allow a more focused look at the oncogenic processes. That is not to say that such scenarios are absent in more common cancers, especially when examined as specific subtypes. However, rare cancers should not simply be ignored because of their rarity or the logistical difficulty of working with them. These anomalies in nature, because of the very fact of their unusual patterns, can hold the key to understanding more common tumors. We term such neoplasms *forme fruste* tumors. *Forme fruste* refers to an attenuated manifestation of disease. We recognize that these tumors are true neoplasms and in many cases, cancers. However, they do not have the genomic baggage and heterogeneity due to genomic instability as seen in common cancers and it is this attenuated genomic phenotype that makes them such tractable and useful targets for genomic research. In this review, we will go over recent sequencing studies of some *forme fruste* tumors which led to discoveries of profound importance.

## Era of next-generation sequencing

Ever since the discovery of DNA and its association with human cancer, scientists and clinicians have dreamt of the possibility to scrutinize it base by base. The ability to sequence DNA, which quickly developed into a robust method by Sanger sequencing [7], was a solid step toward this goal. With the Human Genome Project establishing a map of the human genetic code and rapid advances in computer technology, everything seemed to be in place other than cost and efficiency. Billions of dollars and years of multi-institutional efforts would not make nucleotide sequencing an accessible tool for scientists to ask questions on a regular basis, and the limited resources were not earmarked for the study of rare specimens. The limitations of Sanger sequencing were in the termination of polymerase reactions as well as in the need to separate the products of these reactions by gel or other electrophoretic systems [8]. Additionally, preparation of sequencing libraries was necessary via transformation in *E. coli* or by an incredibly large number of separate PCR reactions. However, with massively parallel sequencing platforms, the first shortcoming was overcome by reversible fluorescent nucleotide addition and imaging (used in Illumina platforms) or through monitoring nucleotide addition via ion detection (used in Ion Torrent platforms (Life Technologies)) both achieved by cyclic manipulation of polymerase or ligase enzymes [9]. Moreover, the second shortcoming was resolved by in vitro library preparation via techniques such as emulsion PCR [10] (Ion Torrent) or bridge PCR on solid surfaces [11, 12] (Illumina). With these improvements, the sequencing cost and time requirements have been vastly reduced. Advancements in bioinformatics and the ability to more readily distinguish signal from noise have also increased the feasibility of large- and small-scale genomic studies. Thus, today, sequencing whole genomes and transcriptomes is more accessible and has become a reality for individual laboratories. We argue that we are in an incredibly exciting era of molecular medicine where a new “molecular microscope” in the form of massively parallel sequencing, also commonly referred to as next-generation sequencing (NGS) or second-generation sequencing, is giving rise to a whole new paradigm for the understanding of human diseases.

**Fig. 1:**
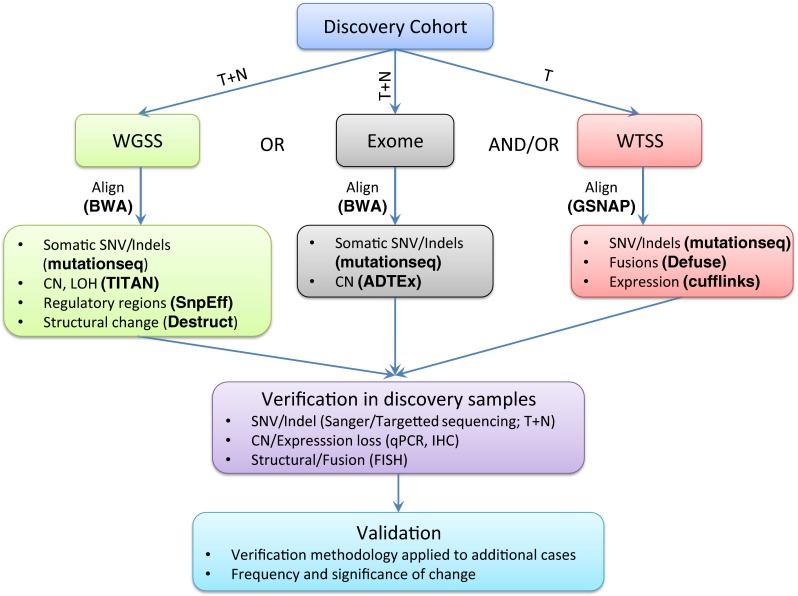
A flowchart of the typical approach to a NGS study to discover novel mutations. Representative bioinformatic programs are in *parenthesis* and in *bold* (further details can be found in [75–81]). For somatic mutations, tumor (T) and matched normal (N) samples, obtained from blood or adjacent normal tissue, are used in whole genome (WGSS) or exome sequencing to look for somatic mutations and copy number changes (CN). Transcriptome analysis (WTSS) of tumor samples will enable assessment of expressed mutations and fusions as well as expression patterns. Confirmation of the NGS findings using a different platform such as Sanger sequencing to eliminate false positives would be the next step. Finally, to understand the frequency of the findings in the disease of interest, analysis on a larger validation cohort of tumor samples should be completed. For hotspot mutations, sequencing; for inactivating mutations, sequencing or immunohistochemistry (IHC); and for fusions, fluorescent in situ hybridization (FISH) could be methods of choice for verification and validation

**Fig. 2:**
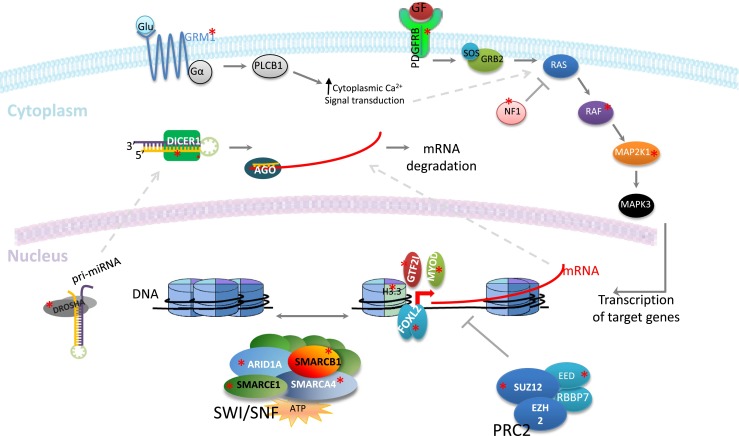
Recent discoveries of mutations in *forme fruste* tumors revealing a broad range of pathways involved. Driver mutations from membrane receptors, to signal transducers, chromatin modifying, and remodeling complexes, as well as transcription factors and microRNA processing factors have been described in a variety of specific tumor pathologies, which may occur at low frequencies in the population. However, such insight, when followed by an understanding of the tumorigenic mechanisms involved, can vastly improve understanding of more common cancers and tumor biology in general. This figure is accompanied by Table 2 which includes a list of such tumors as well as the indicated mutations (marked by *asterisks*)

We will discuss recent attempts to study *forme fruste* tumors using NGS. The general approach and representative bioinformatic tools employed in such studies are summarized in Fig. 1. Specific *forme fruste* tumor types will be discussed that exemplify the impact of such studies in three categories: a better definition of an already known disease, establishment of new disease subtypes, and development of novel insights into oncogenic mechanisms. Table 1 includes a more comprehensive summary of discoveries in *forme fruste* tumors with a focus on recent NGS findings and their study designs. In addition, Fig. 2, accompanied by Table 2, demonstrates the broad range of pathways affected in such tumors. Lastly, we will go over some of the challenges and scenarios where the study of certain *forme fruste*-like tumors shows that their genomic behavior is not always straightforward. In addition to the broad array of discoveries made, the small number of cases used in each successful discovery process is perhaps noteworthy (Table 1) with the single case of endometrial stromal sarcoma where, as described below, sequencing led to a diagnostic and formal disease reclassification.

**Table 1:** A list of mutations in pathologically specific tumors with a focus on recent NGS discoveries, including discovery methodology, and validation results

Disease	Mutation	Discovery methodology	Discovery cohort size	Frequency in validation cohort (number of cases)	Reference
Congenital fibrosarcoma	*ETV6-NTRK3* (fusion)	FISH positional cloning, RACE	4 primary tumors grown in culture	Same as discovery, 100 % (3)	[95]
Granulosa cell tumor	*FOXL2* (hotspot)	WTSS	4 tumors	97 % (89)	[18]
CCC	*ARID1A* (loss)	WTSS	19 tumors	46 % (119)	[44]
		Exome capture, NGS	8 tumor enriched samples (magnetic beads)	57 % (42)	[45]
Epithelioid hemangioendothelioma	*WWTR1-CAMTA1* (fusion)	WTSS	1 case with t(1;3)(p36;q25), snap	87–89 % (45–47)	[25]
		FISH positional cloning	17 FFPE	Same as discovery, 100 % (17)	[28]
Nonepithelial ovarian tumors	*DICER1* (hotspot)	WTSS and exome capture	14 samples, 5 with matched normal	20 % (145)	[59]
EWSR1-ETS-negative small round cell bone sarcoma	*BCOR-CCNB3* (fusion)	WTSS	4 tumors	4 % of fusion negative sarcomas (594)	[35]
High-grade endometrial stromal tumors	*YWHAE-FAM22* (fusion)	WTSS	1 cell line	100 % (12)	[32]
Chondrosarcoma	*COL2A1* (inactivating)	Exome	49 tumors with matched normal	44 % (26)	[111]
Solitary fibrous tumor	*NAB2-STAT6* (fusion)	Exome	17 tumors with matched normal	55 % (29)	[112]
NF2-negative familial multiple spinal meningioma	*SMARCE1* (loss)	Exome	3 unrelated individuals	33 % (6)	[54]
Chondroblastoma	*H3F3B* (hotspot)	WGSS	6 tumor/normal matched	95 % (77)	[69]
Giant cell tumors of bone	*H3F3A* (hotspot)	Sanger sequencing		92 % (53)	
DIPG/pediatric GBM	*H3F3A* (hotspot)	WGSS	7 with matched normal	78 % (43)	[64]
	*H3F3A* (hotspot)	Exome	48, 6 with matched normal	36 % (42)	[65]
	*ATRX* (loss)			Sequencing 33 % (42), IHC 35 % (113)	
	*ACVR1* (hotspot)	WGSS, exome, WTSS	42 HGG tumors with matched normal	32 % of DIPG(80)	[100]
		WGSS, exome, microarray	36 frozen tumors and matched normal	20 % (25)	[101]
		WGSS, exome	26 tumors and matched normal	21 % (26)	[102]
		Exome, WTSS	39 tumors with matched normal	13 % (same as discovery cohort)	[103]
Glomus tumor	*MIR143-NOTCH* (fusion)	WTSS	3 tumors	64 % (33)	[88]
Familial infantile myofibromatosis	*PDGFRB* (hotspot)	Exome	11 germline (familial, 9 families)	89 % (9 families)	[84]
		Exome, WTSS	2 germline (exome), 1 tumor (WTSS)	100 % of familial (8), 0 % of simplex (5)	[85]
SCCOHT	*SMARCA4* (loss)	WGSS, Exome	13 tumor/normal pairs	82 % (17)	[49]
		Exome	6 tumor/normal pairs (familial, 3 families)	90 % (20)	[50]
		Exome	12 tumor/normal pairs	88 % (43)	[51]
		Sanger sequencing, IHC	2 FFPE cases	None	[52]
Chondromyxoid fibroma	*GRM1* (fusion)	SNP Arrays, WGSS, WTSS	8 tumors , 2 with both WGSS and WTSS data	RT-PCR 90 % (20)	[82]
Biphenotypicsinonasal sarcoma	*PAX3-MAML3* (fusion)	WTSS	1 tumor	96 % (25)	[83]
Maxillary ameloblastoma	*SMO* (hotspot)	WTSS	2 FFPE cases	82 % (11)	[39]
Mandibular ameloblastoma	*BRAF* (hotspot)			69 % (13)	[39]
MPNST	*SUZ12, EED* (inactivating)	WGSS or exome	8 tumors, 5 with matched normal	SUZ12 in 26 % (42)	[86]
		Exome, WTSS	15 tumors with matched normal	SUZ12 in 49 % and EED in 38 % (37)	[87]
PLGA	*PRKD1* (hotspot)	WTSS, Exome	6 tumors, 3 with matched normal	72 % (53)	[89]
ALK-negative inflammatory myofibroblastic tumor	*ALK, ROS1* and *PDGFRB* (fusions)	Targeted NGS	1 FFPE (tumor)	73 % (11)	[93]
Thymoma	*GTF2I* (hotspot)	Exome	28 tumors with normal	82 % type A, 74 % type AB (274)	[96]
Cortisol-producing adrenal tumors	*PRKACA* (hotspot)	Exome	25 tumors and matched normal	21 % (63)	[97]
		Exome	8 tumors with matched normal	53 % (57)	[104]
ERMS	*MYOD1* (hotspot)	Exome and WTSS	20 tumors (8 with both WTSS, exome)	10 % (104)	[70]
Brainstem gliomas	*PPM1D* Exon 6 truncation, activating	Exome	26 tumors with matched normal	37.5 % of H3F3A harboring BSG (24)	[98]
Diffuse gastric carcinoma	*RHOA* (hotspot, GOF)	Exome	30 tumors with matched normal	25 % (87)	[99]
AITL	*RHOA* (hotspot, inactivating)	Exome, WTSS, aCGH	5 tumor/normal pairs, 4 tumor only	53 % (45)	[105]
		Exome	12 PTCL tumor/normal pairs (3 AITL)	Allele-specific PCR mutation assay 67 % (43 AITLs)	[107]
		Exome	3 tumor/normal pairs	71 % (72)	[108]
PMBCL	*PTPN1* (inactivating)	WGSS, WTSS	10 tumors, 2 with matched normal	22 % (77)	[106]
Ewing sarcoma	*STAG2* (inactivating)	WGSS	6 tumors with matched normal	IHC 12 % (154)	[90]
		Exome	26 matched tumors, 66 tumor only, 11 lines	15 % (73), IHC	[91]
		WGSS	112 tumors with matched normal	Capture sequencing 13 % (199)	[92]
		SNP array	1 cell line, U138MG	IHC 21 % (53)	[94]
IGHV4-34+ HCL	*MAP2K1* (hotspot)	Exome	10 tumors, 6 with matched normal	48 % (21)	[109]
Familial schwannomatosis	*LZTR1* (inactivating)	Targeted deep sequencing	16 unrelated cases, germline mutations	75 % (12)	[110]

References: [18, 25, 28, 32, 35, 39, 44, 45, 49–52, 54, 64, 65, 69, 70, 82–112]

*CCC* clear cell carcinoma of the ovary, *DIPG* diffuse intrinsic pontineglioma, *GBM* glioblastoma multiforme, *SCCOHT* small-cell carcinoma of the ovary of the hypercalcemic type, *MPNST* malignant peripheral nerve sheath tumor, *ERMS* embryonal rhabdomyosarcoma, *PLGA* polymorphous low-grade adenocarcinoma of salivary gland, *AITL* angioimmunoblastic T cell lymphoma, *PMBCL* primary mediastinal B cell lymphoma, *GOF* gain of function, *FISH* fluorescent in situ hybridization, *RACE* rapid amplification of cDNA ends, *WGSS* whole genome shotgun sequencing, *WTSS* whole transcriptome shotgun sequencing, *IHC* immunohistochemistry, *aCGH* array comparative genomic hybridization

**Table 2:** List of mutations indicated in Fig. 2

Pathway	Target	Mode of dysregulation	Tumor example
SWI/SNF	*SMARCA4*	Inactivated	SCCOHT, medulloblastoma, Burkitt’s lymphoma, NSCLC
	*SMARCB1*	Inactivated	Rhabdoid tumors, epithelioid sarcoma, CRINET, SBC, schwannomatosis, renal medullary carcinoma, gastrointestinal neoplasms
	*SMARCE1*	Inactivated	Familial multiple spinal meningiomas
	*ARID1A*	Inactivated	CCC, EC, GC, neuroblastoma
Histone 3.3	*H3F3A*	Hotspot	GCBT, DIIPG
	*H3F3B*	Hotspot	Chondroblastoma
Transcription factors	*GTF2I*	Hotspot	Thymoma
	*MYOD1*	Hotspot	ERMS
	*FOXL2*	Hotspot	GCT
PRC2 complex	*SUZ12*	Inactivated	MPNST
	*EED*	Inactivated	MPNST
miRNA processing	*DICER*	Hotspot	NEOC, e.g., SLCT
	*DROSHA*	Hotspot	WT
RTK signal transduction	*PDGFRB*	Hotspot	IMT
		Fusion	FIM
	*NF1*	Inactivated	MPNST
	*RAF*	Hotspot	M. Ameloblastoma
	*MAP2K1*	Hotspot	IGHV4-34^+^ HCL
GPCR	*GRM1*	Fusion	Chondromyxoid fibroma

*RTK* receptor tyrosine kinase, *SCCHOT* small-cell carcinoma of the ovary of the hypercalcemic type, *NSCLC* nonsmall-cell lung cancer, *CRINET* cribriform neuroepithelial tumor, *SBC* sinonasal basaloid carcinoma, *RMC* renal medullary carcinomas, *CCC* clear-cell carcinoma of the ovary, *EC* endometriod carcinoma of ovary, *GC* gastric cancer, *GCBT* giant cell bone tumor, *DIPG* diffuse intrinsic pontineglioma, *ERMS* embryonal rhabdomyosarcoma, *GCT* granulosa cell tumor, *MPNST* malignant peripheral nerve sheath tumor, *NEOC* nonepithelial ovarian cancer, *SLCT* Sertoli-Leydig cell tumor, *WT* Wilm’s tumor, *IMT* inflammatory myofibroblastic tumor, *FIM* familial infantile myofibromatosis, *M. Ameloblastoma* mandibular ameloblastoma, *HCL* hairy cell leukemia

## Better definition of an existing pathology

Pathognomic mutations allow a more specific definition of a previously established histological diagnosis. Below, we will discuss the cases of the *FOXL2* mutation in adult-type granulosa cell tumors (GCTs) as well as the *WTR1-CAMTA1* fusion in epithelioid hemangioendotheliomas (EHEs).

### Granulosa cell tumors and *FOXL2* mutations

GCTs are rare, constituting only about 5 % of ovarian tumors [13]. Until 2009, the molecular biology of this tumor remained a mystery and hence, there was limited success in development of therapeutics for aggressive cases [14]. There are two subtypes of this tumor: adult-type and juvenile. These have similar biomarker profiles but occur in different age groups and have different histopathological features [13]. Adult-type GCTs fit the concept of a *forme fruste* cancer: cytogentics had shown a more stable genome compared to other ovarian tumors [15], and the tumor subtypes have a consistent pathological presentation with cells that have maintained some levels of differentiation expressing follicule-stimulating hormone receptors and inhibin [16]. There were no associations between expression of common oncogenes and tumor suppressors, such as *MYC*, *TP53*, *ERBB2*, or *RAS* family and outcomes in GCTs [17]. Based on these facts, Shah et al. reasoned that with sequencing of very few adult-type GCT cases, rather than the massive sample sets needed for the more common genetically complex tumors, considerable insight into the biology of GCTs could be attained. Thus, only four samples of adult-type GCTs were used for whole transciptome sequencing as a discovery cohort and 11 other ovarian tumors were sequenced as a comparative cohort [18]. After alignment and removal of previously reported germline insertions and deletions, there were between 289 and 495 somatic nonsynonymous variants in the GCTs. Genes with mutations in at least three of the four cases that were not mutated in the comparative cohort were considered for further follow-up [18]. The only potential mutation found in all four cases was a C134W mutation in *FOXL2* [18]. The resulting mutant protein was still expressed in GCTs as observed by immunohistochemistry in cases with apparent homozygosity (likely through loss of the normal allele), meaning most likely there was a gain/switch of function. In a validation cohort of an additional 95 sex-cord stromal ovarian tumors, specificity and sensitivity of the C134W mutation in adult-type GCTs was established [18]. This study was significant in three aspects: it was the first time that a consistent genetic event was associated with GCTs, the first time *FOXL2* had been indicated to have an oncogenic role in any tumor, and the first example of a novel disease-defining pathognomonic driver mutation being discovered using massively parallel sequencing.

Although FOXL2 is known to be critical for the development of ovaries and is one of the early differentiation markers [19], somatic mutations in this transcription factor had not been linked to pathology before this study. The diagnostic implications of the C134W mutant *FOXL2* have already become apparent [20, 21], and some studies have since looked at potential mechanistic pathways. It has been suggested that the hotspot FOXL2 mutation might have very particular effect in a specific context: the mutant FOXL2 reduces the expression of gonadotropin-releasing hormone (GnRH) receptor and limits the GnRH-induced apoptosis seen in normal human granulosa cells [22]. This finding shows that tissue-specific pathways may be the bottlenecks that limit driver mutations that can arise in a specific cell of origin. Furthermore, the mutant FOXL2 has also been suggested to be less stable because of increased phosphorylation via GSK3β and MDM2-mediated ubiquitination and proteasome degradation [23]. Hence, inhibition of GSK3β has already been identified as a therapeutic target that stabilizes mutant FOXL2 and this stabilization may in turn lead to increased apoptosis.

### *WWTR1-CAMTA1* in EHE

Another example of a disease-defining mutation came with EHE, a rare tumor that can present diagnostic challenges. The tumor is a vascular sarcoma with epithelial-looking cells that show vascular differentiation with positivity for platelet endothelial cell adhesion molecule and CD34 [24]. Through the use of a single index case, Tanas and colleagues were able to identify a fusion of *WWTR1* to *CAMTA1* and establish it as a specific event in EHE [25]. In a validation cohort of 47 cases, they showed that rearrangements of the involved genes happened 87–89 % of the time whereas none of 118 cases of other vascular tumors showed these rearrangements [25]. Part of the success in identifying this pathognomonic fusion was due to the already known recurrent translocation involving chromosomes 1 and 3 in EHE [26, 27]. This meant that during the bioinformatic analysis, focus was limited to the predicted fusions involving genes on these chromosomes. Simultaneously, another group was also able to use the more traditional method of fluorescent in situ hybridization (FISH) positional cloning in 17 cases of EHE and also discovered the fusion partners *WWTR1* and *CAMTA1* [28].

*WWTR1* (also known as *TAZ*) encodes a transcriptional coactivator containing the WW domain. This domain, which is named as such because of the two conserved tryptophans, mediates specific protein-protein interactions and has been implicated in Hippo signaling, a critical pathway in regulating organ size and keeping proliferation in check. *WWTR1* is phosphorylated by lats tumor suppressor kinases (*LATS2*) which are key components of Hippo signaling and this in turn leads to binding by 14-3-3 proteins which lead to cytoplasmic localization and hence inactivation of *WWTR1* [29]. Interestingly, in the EHE fusion, the 14-3-3 binding domain of WWTR1 is maintained; however, one of the critical LATS2 phosphorylation sites, namely Ser311, is lost. This perhaps could render the fusion protein partly resistant to inhibition by Hippo signaling. CAMTA1 is a transcriptional regulatory protein with the capacity to bind DNA. Because WWTR1 has no known DNA-binding motifs and since the DNA-binding domain of CAMTA1 is maintained, the new fusion protein might giveWWTR1 a new ability to bind DNA [25].

## Establishment of new classification/subtype

In addition to identifying pathognomonic mutations by deep sequencing a few samples, sequencing studies of *forme fruste* tumors have led to new classification and subtype establishments. Three examples are discussed as follows.

### *YWHAE* fusions in high-grade endometrial stromal sarcoma

Endometrial stromal sarcoma (ESS) is a malignancy of the uterus that had been previously linked with recurrent fusions: the fusion of *JAZF1*, a transcriptional repressor, with members of the polycomb complex including *SUZ12*, *PHF1*, and *EPC1* [30, 31]. Yet, there remained a subset of ESS tumors, often with a higher histologic grade, that could not be demonstrated to carry fusions involving these genes. Lee and colleagues thus decided to look in depth at the genomics of such cases and ended up discovering recurrent fusions involving *YWHAE* and the *FAM22* family [32]. As in earlier work, Lee and colleagues first drew on results from cytogenetic studies and noted a recurrent t(10;17)(q22;p13). Similar to the case with the *WWTR1-CAMTA1* fusion in EHEs, this karyotype information greatly aided the analysis of the next-generation sequencing data such that, by the use of just one sample, they were able to focus on the *YWHAE-FAM22A* translocation event, later showing that in cases missing this particular fusion, *YWHAE* was fused to homologs of *FAM22A* such as *FAM22B* [32]. This work established a new entity of higher-grade endometrial stromal sarcomas with a molecular defining feature that distinguishes them from other endometrial stromal sarcomas [33]. In fact, soon after the discovery of this novel fusion, the World Health Organization incorporated the presence of *YWHAE-FAM22* translocations into the classification of endometrial stromal sarcomas [34].

### *BCOR-CCNB3* bone sarcoma

An exemplary case of NGS defining a new pathology came through the study of peculiar small round cell bone sarcomas that lacked the *EWSR1-ETS* fusions of the top candidate in the differential diagnosis, Ewing sarcoma. Four index cases were used for RNA-seq with fusion analyses, and out of these strong evidence for fusion transcripts was seen in two cases: one that had an atypical Ewing fusion of *FUS*-*FEV* and another with a completely novel fusion of exon 15 of *BCOR* to exon 5 of *CCNB3* [35]. The authors then carried out a comprehensive RT-PCR screening of 594 sarcomas lacking fusions classically sought in diagnostics laboratories. They were able to identify an additional 24 cases of sarcomas with the *BCOR-CCNB3* fusion. Microarray expression profiling of ten such cases showed that these tumors had a different profile than other tumors in the differential diagnosis such as Ewing sarcoma, and hence, a whole new bone sarcoma was established [35]. *BCOR* is thought to encode a ubiquitously expressed protein with a role in repression of transcription through epigenetic mechanisms and in mesenchymal stem cell function [36]. On the other hand, *CCNB3* expression is restricted to testis and the encoded protein is a cyclin expressed during spermatogenesis [37]. The ectopic expression of CCNB3 as a result of the fusion event could be the driver of oncogenesis in this novel sarcoma. Indeed, expression of both the truncated and *BCOR* fused *CCNB3* in fibroblast lines leads to increased proliferative capacity [35].

### Maxillary versus mandibular ameloblastomas

Another recent study established that ameloblastomas, rare benign tumors of the jaw thought to originate from ameloblasts [38], have distinct recurrent mutations depending on whether they arise in the maxilla versus the mandible. The maxillary ameloblastomas harbor a *SMO* hotspot mutation, and the mandibular tumors have *BRAF* hotspot mutations [39]. Although ameloblastomas are benign and rare tumors, this study emphasizes the mutational heterogeneity of histologically indistinguishable tumors depending on their location and highlights the significance of molecular classification. As mutant BRAF, commonly seen in melanomas, can be targeted with new therapies, this finding also has immediate therapeutic implications. Associations of tumor location and defining mutations have also been identified, for instance, in mengiomas: those that arise in the lateral and posterior regions bear *NF2* mutation whereas those in the anterior and medial regions do not [40]. Even in rare tumors with seemingly distinctive histology, there exist subsets defined by specific molecular aberrations. The new disease subclassifications thereby identified may be of great significance for development and application of targeted therapeutics.

## Insights into cancer mechanisms

The study of rare tumors has also expanded our knowledge about cancer pathways. We will focus on recent findings of recurrent mutations in chromatin remodelers, microRNA processors, and histones.

### SWI/SNF mutations in ovarian epithelial tumors and meningiomas

Clear-cell ovarian carcinomas are the second most common type of ovarian cancer [41] and until 2010 were not very well studied despite evidence of relative genomic stability [42, 43]. With whole transcriptome sequencing/exome sequencing, recurrent mutations in *ARID1A*, a member of the already established SWI/SNF chromatin remodeling complex were found [44, 45]. The mutations were spread across the *ARID1A* gene and led to its inactivation, thus suggesting that this gene may function as a tumor suppressor. Although other core members of the SWI/SNF complex had been linked to cancer previously (*SMARCB1* and *SMARCA4* are known to have lost expression in a variety of tumors), this study showed that noncanonical members of the SWI/SNF complex could also play important roles in tumorigenesis. Furthermore, lack of evidence for mutations in other members of the complex hinted at a context-specific tumor suppressor role for the individual members of the SWI/SNF complex. Additionally, Wiegand and colleagues also showed that the mutation was present in precursor atypical endometriotic lesions of the tumor, and thus was likely an early driver of ovarian clear-cell carcinoma. *ARID1A* mutations were later found in a variety of other more common types of cancer including gastric adenocarcinomas [46] and colorectal cancers [47].

Small-cell carcinoma of the ovary of the hypercalcemic type (SCCOHT) is another rare but genetically stable tumor [48] that was discovered to have abnormalities in the SWI/SNF complex. In this case, the core enzymatic unit of the protein complex, *SMARCA4*, was mutated in an inactivating fashion in the majority of cases, and almost all tumors of this specific diagnosis stained negatively for *SMARCA4*’s protein product BRG1 [49–52]. Although mutations in *SMARCA4* have been described in more common cancers such as lung adenocarcinomas [53], they occur in a fraction of cases and are not the obvious drivers of oncogenesis. The studies in SCCOHT with loss of *SMARCA4* in almost all cases emphasized the driver role of *SMARCA4* loss. Another example of a critical driver role of SWI/SNF mutations came through the NGS study of familial multiple spinal meningiomas [54]. In familial cases, which tested negative for previously described *NF2* or *SMARCB1* mutations, germline *SMARCE1* mutations were identified through exome sequencing. Again, the protein was lost in the tumor samples but not in normal tissue, thus suggesting a classic Kundson biallelic inactivation and a tumor suppressor role of *SMARCE1*.

It should be noted that the reason for disease specificity of SWI/SNF member mutations and indeed the steps in tumorigenesis associated with their loss are not clear. SWI/SNF is thought to regulate the expression of many genes and interacts with many critical cancer pathways from cell cycle regulation to hedgehog and Wnt signaling. Indeed, it has been suggested that perhaps the remaining complex, which still assembles without the mutated members, might act as an oncoprotein and drive tumorigenesis [55]. Thus, much is still to be clarified in this area; however, since the establishment of the association of the SWI/SNF complex with cancer in rather rare entities, we know today that about 20 % of all cancers have mutations in this complex [56]. However, the impact of these mutations is by and large yet to be established.

### MicroRNA processing mutations in nonepithelial ovarian tumors

Given abnormalities in microRNA levels in certain cancer, it was thought that the genes encoding proteins involved in microRNA processing might also be of significance in oncogenesis. Germline mutations in *DICER1* were identified in the rare familial pleuropulmonary blastoma–family tumor and dysplasia syndrome [57]. However, the first evidence for somatic oncogenic mutations of *DICER1* came from the study of nonepithelial ovarian tumors [58]. Recurrent somatic hotspot mutations in *DICER1* were identified across nonepithelial ovarian tumor types and were most predominantly seen in Sertoli-Leydig cell tumors [59]. Although low expression of *DICER1* has been previously associated with worse prognosis in breast cancer [60] and ovarian tumors [61], the study of these nonepithelial ovarian tumors changed the paradigm as for the first time it was found that a hotspot genetic aberration in *DICER1* can drive cancer through the combination of loss of one allele and a functionally deficient protein, this is an aberration of the classic two-hit hypothesis [59]. In actuality, *DICER1* in nonepithelial ovarian tumors does not fit traditional tumor suppressor or oncogene models. Rather, there seems to be a mix of the two models involved in tumorigenesis. There is an inherited inactivation of one copy of the genes, and the remaining allele is not totally inactivated somatically, which would be lethal in most cells rather is hypomorphic via hotspot mutations (Fig. 2). The hotspot mutations are found in the RNaseIIIb metal-binding site, reducing RNaseIIIb activity and leading to a global loss in the processing of mature 5p microRNAs but maintenance of 3p processing [62]. Later studies showed that oncogenic mutations in *DROSHA*, another microRNA processing gene, and associated global microRNA changes also occur in Wilm’s tumor [63]. Therefore, processors of microRNA represent another family of cancer-associated proteins and *forme fruste* tumors were significant in this realization.

### Histone mutation in bone and central nervous system tumors

Another prime example of insights into cancer biology comes from the identification of mutations in histones in *forme fruste* tumors. Mutations in *H3F3A*, which encodes histone 3.3, were identified in pediatric diffuse intrinsic pontinegliomas (DIPGs) [64] and pediatric glioblastomas [65]. Histone 3.3 is a member of the histone 3 family which is associated with active chromatin and is incorporated into chromatin throughout the cell cycle [66–68]. Interestingly, an independently regulated gene named *H3F3B* also seemingly encodes the same histone 3.3 protein; however, mutations in this gene were not identified in DIPGs or glioblastomas. In a seminal study, Behjati et al. described *H3F3A* driver mutations in another tumor type: chondroblastomas [69]. Additionally, they also discovered novel *H3F3B* mutations in giant cell tumors of bone [69]. Chondroblastomas and giant cell tumors of bone have similarities such as clinical presentation in the bone epiphysis and the presence of large numbers of osteoclastic giant cells; however, they tend to affect different age groups and have different clinical outcomes. As mentioned, the two genes encode the same protein, yet Behjati et al. showed a clear predilection toward *H3F3A* or *H3F3B* depending on tumor type. Since there is no expression difference between these genes in giant cell bone tumor versus chondroblastomas, temporal expression, for instance at the time of tumor formation, is a possibility suggested by the authors [69]. The above studies were of great value shifting the focus from histone modifying complexes to histones themselves and showing that mutations in histones can be driver mutations.

### Concluding remarks

It should be noted that rare tumors with homogenous clinical behavior are not always easy to study, and the examples used above are success stories that have benefitted from the relative ease of interpreting NGS results when the tumors are truly simple genomically. Embryonic rhabdomyosarcomas have clinical and morphologic features of *forme fruste* tumors but ended up revealing a complex genome with various tumorigenic mechanisms identified in different cases, unlike the more consistent drivers seen in the tumor types described above [70, 71]. Similarly, our own group’s study of epithelioid sarcoma has revealed that despite its unique and consistent pathology and biology, this tumor has a relatively complex genome.

Yet, as a whole, *forme fruste* tumors have been particularly informative in deep sequencing studies, expanding on our knowledge of cancer biology in a resource-efficient manner. Here, we have cited several successful examples of recent findings that have lead to the discovery of pathognomonic mutations, the establishing new subtypes and classifications, such as the case of high-grade ESS, and providing insight into mechanisms of cancer formation such as findings of SWI/SNF and microRNA processing gene abnormalities. This is not to say that such discoveries are not possible in more common, genetically complex cancers, but in *forme fruste* tumors, the reduced complexities in the genome allows for identification of driver oncogenic events with the use of very few samples. Part of the reason for success in studying these tumors can also be attributed to the fact that they have tended to be understudied and not so much is known about them. However, their rarity comes with the challenge of a lack of banked samples appropriate for the nucleic acid extractions needed for deep sequencing. Recent advancements in sequencing technologies mean that formalin-fixed paraffin-embedded tissues can now be also used for deep sequencing, and hence, some of the challenges in studying *forme fruste* tumors are already being overcome [39, 72].

## Beyond next-generation sequencing

The focus of this review has been on next-generation sequencing and its role as a molecular microscope helping define tumors in a new way. However, sequencing technologies and associated analytic capacities are advancing at a rapid rate. The ability to study clonal evolution and diversity, which has been successfully utilized in the breast cancer field for instance [73], can be of great value if applied to rare conditions to see the degree of genomic heterogeneity in these seemingly simpler tumors (which should have a more interpretable signal-to-noise ratio for subclone tracking). Additionally, tools such as single-cell RT-PCR and single-cell next-generation sequencing that are in development and reviewed elsewhere [74] can reveal a whole new window on intratumoral heterogeneity, and *forme fruste* tumors, in particular biphasic cancers like synovial sarcoma, can again provide models that may well prove easier to study. We believe that with all these developing methodologies, rare tumors can be a source of breakthroughs that give clearer answers at lower cost, with fewer samples needed to make discoveries.
